# Inferring spatially varying animal movement characteristics using a hierarchical continuous‐time velocity model

**DOI:** 10.1111/ele.14117

**Published:** 2022-10-18

**Authors:** Ionut Paun, Dirk Husmeier, J. Grant C. Hopcraft, Majaliwa M. Masolele, Colin J. Torney

**Affiliations:** ^1^ School of Mathematics and Statistics University of Glasgow Glasgow UK; ^2^ Institute of Biodiversity, Animal Health & Comparative Medicine University of Glasgow Graham Kerr Building Glasgow UK

**Keywords:** animal movement, Bayesian inference, Serengeti wildebeest, spatial ecology

## Abstract

Understanding the spatial dynamics of animal movement is an essential component of maintaining ecological connectivity, conserving key habitats, and mitigating the impacts of anthropogenic disturbance. Altered movement and migratory patterns are often an early warning sign of the effects of environmental disturbance, and a precursor to population declines. Here, we present a hierarchical Bayesian framework based on Gaussian processes for analysing the spatial characteristics of animal movement. At the heart of our approach is a novel covariance kernel that links the spatially varying parameters of a continuous‐time velocity model with GPS locations from multiple individuals. We demonstrate the effectiveness of our framework by first applying it to a synthetic data set and then by analysing telemetry data from the Serengeti wildebeest migration. Through application of our approach, we are able to identify the key pathways of the wildebeest migration as well as revealing the impacts of environmental features on movement behaviour.

## Graphical abstract

We develop a Bayesian hierarchical modelling framework that allows us to infer the landscape level characteristics of observed animal movement patterns. We apply our framework firstly to synthetic movement data to demonstrate its accuracy and effectiveness, and then to GPS telemetry data from a long‐term study of the Serengeti wildebeest migration. Our empirical data analysis reveals the migratory pathways of the Serengeti wildebeest and shows how specific regions associated with high human presence significantly alter movement behaviours.

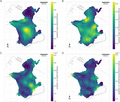

## INTRODUCTION

Increasingly, animals are moving through human‐altered landscapes (Tucker et al. 2018). Infrastructure, growing human populations and artificial boundaries, such as fences or roads, are disrupting animal movement patterns (Doherty et al., 2021; Løvschal et al., 2017; Wittemyer et al. 2008) and consequently, many far‐ranging or migratory species are in decline (Campbell et al., 2021; Harris et al., 2009; Studds et al., 2017; Wilcove & Wikelski, 2008). In order to effectively protect these species, it is essential to understand how animals respond to environmental features and disturbances and to identify the areas, such as migratory corridors, stop‐over sites or foraging grounds that are vital for the survival of a species.

In recent years, there has been a rapid advance in our ability to collect high frequency data on the movement and behaviour of animals (Brown et al., 2013; Kays et al., 2015; Wilmers et al., 2015). Allied with the increase in data availability has been the development of statistical models (Hooten et al., 2017) that infer key characteristics of movement and identify the drivers of observed movement patterns, one of the core aims of movement ecology (Nathan et al., 2008). A fundamental component in the statistical analysis of movement has been the random walk model (Codling et al., 2008; Fagan & Calabrese, 2014; Kareiva & Shigesada, 1983). Using both continuous and discrete time formulations, this approach has been employed to detect different behavioural modes, such as encamped or exploratory, in movement data (Morales et al., 2004), to refine home range estimates based on the autocorrelation present in trajectories (Fleming et al., 2015), to detect spatially or temporally shifting migration routes (Gurarie, Cagnacci, et al. 2017) and to evaluate the role of social interactions in driving movement decisions (Haydon et al., 2008; Torney, Lamont, et al. 2018).

Examining the landscape level drivers of movement has typically employed parametric functions of environmental covariates via hidden Markov models (Langrock et al., 2012) or step selection functions (Avgar et al., 2016; Thurfjell et al., 2014). Recently, non‐parametric approaches have been proposed that allow continuous and time‐varying movement parameters to be incorporated into models (Michelot et al., 2021; Torney et al., 2021). An important next step in the development of these models is to identify the underlying spatially varying factors that influence animal movement. To do so requires flexible, data‐driven models that are able to capture complex spatial features and the nonlinear behavioural responses they elicit, as well as being able to efficiently process the large amounts of data required for accurate inference.

In this work, we present a hierarchical spatial model based on multilevel Gaussian processes (GPs) (Heinonen et al., 2016; Rasmussen & Williams, 2006). We employ a velocity‐based movement model which is linked to an underlying latent spatial field via the introduction of a novel non‐stationary covariance matrix. While previous works (Hooten & Johnson, 2017; Torney et al., 2021) have also applied hierarchical GPs to animal movement, the novelty of our approach is that by linking the velocity‐based movement model to latent spatial fields we are able to reveal the persistent, spatially varying movement characteristics of mobile animal populations based on positional data collected from multiple individuals. To enable efficient inference of data sets consisting of potentially millions of data points, we employ Bayesian variational learning (Blei et al., 2017) a novel approach in this context that replaces traditional computationally expensive sampling‐based inference with fast stochastic optimisation. For data set sizes of over one million location observations, we show that model fitting takes in the order of hours yet still provides full posterior distributions over the latent fields.

In what follows, we firstly present further theoretical background on non‐stationary GPs. We next describe the computational inference methodology we employ to fit the model to data and provide two example studies. In the first, we generate a synthetic data set with known properties that we infer with our framework. In our second case study, we apply the framework to telemetry data collected over a period of 6 years from a long‐term study of the Serengeti wildebeest migration.

## METHODS

### Background

Within statistical ecology, Gaussian random fields are a popular tool for the modelling and analysis of spatial data (Banerjee et al., 2003; Rue et al., 2009). As opposed to semi‐parametric approaches, such as splines or radial basis functions, a random field models a two‐dimensional surface (representing a latent field or spatially correlated residuals) as a realisation of a stochastic process (Gelfand & Schliep, 2016). If every finite collection of random variables that form this stochastic process has a multivariate normal distribution, then the random field is a Gaussian random field, or GP.

As all linear stochastic differential equations (SDEs) can be expressed as GPs with an appropriate covariance structure (Särkkä et al., 2013), all random walk movement models that can be formulated as a linear SDE are also equivalent to GPs (Hooten & Johnson, 2017; Torney et al., 2021). Hence, linking spatial Gaussian random fields with a continuous‐time movement model involves linking one GP with another and is an example of multi‐layered GP regression. Inference with multi‐layer GPs is an active area of research in the machine learning community, and several different approaches have been employed. One approach is to use the outputs from multiple low level GPs to define the covariance structure of a high‐level GP (Heinonen et al., 2016) leading to a non‐stationary stochastic process at the highest level. This method can be used to model data that have characteristics, such as autocorrelation or variance, that vary over time or space. It is this approach that we adopt in this work to learn multiple latent spatial fields that define the parameters of a continuous‐time velocity model of animal movement (Johnson et al., 2008). The spatial fields are therefore the lower level GPs which provide the parameters of a covariance function of a higher level GP. These parameters have a clear ecological interpretability, representing the directional persistence and average speed of individuals at each location of the landscape.

In standard GP regression, we have some input locations x and some outputs y are observed. We assume $y_{i}=f\left(x_{i}\right)+\varepsilon_{i}$ for some unknown function f with added white noise $\varepsilon_{i}$, where $x_{i}$ and $y_{i}$ are elements of x and y_,_ respectively. Unlike a parametric approach, where the focus is on parametric representations for the function f, a GP adopts a non‐parametric approach by assuming a multivariate Gaussian prior over possible functions and, once data have been observed, producing a posterior distribution over $f\left(x\right)$ that is consistent with the observed values of y. The aim of GP regression is therefore to infer a distribution over functions given the data, $p\left(f|x,y\right)$, and then to use this to make predictions given new input locations $x^{*}$, i.e. to compute

(1) $$p\left(y^{*}|x^{*},x,y\right)=\int p\left(y^{*}|f,x^{*}\right)p\left(f|x,y\right)df.$$

Calculating the conditional probabilities, $p\left(f|x,y\right)$ and $p\left(y^{*}|x^{*},x,y\right)$, is made possible by the specification of a GP prior on the function f. We have assumed that our prior distribution over possible functions is defined as

(2) $$f\left(x\right)∼\mathrm{GP}\left(m\left(x\right),\;k\left(x,x^{\prime }\right)\right),$$

where $m\left(x\right)=E\left[f\left(x\right)\right]$ is the mean function and $k\left(x,x^{\prime }\right)=E\left[\left(f\left(x\right)-m\left(x\right)\right)\left(f\left(x^{\prime }\right)-m\left(x^{\prime }\right)\right)\right]$ defines the covariance function (or kernel). For standard GP regression, the key challenge for fitting a model to data is appropriately specifying the covariance kernel, $k\left(x,x^{\prime }\right)$. If $k\left(x,x^{\prime }\right)=k\left(|x-x^{\prime }|\right)$, i.e. the covariance value depends only on the distance between two locations and not their absolute location, then this is termed a stationary covariance kernel. One choice of stationary covariance kernel is the exponential kernel defined as

(3) $$k\left(|x_{i}-x_{j}|\right)=\sigma \exp\left(-\frac{|x_{i}-x_{j}|}{\tau }\right),$$

where the kernel hyperparameters σ and τ control the amplitude and correlation length of the latent function f. Typically, the hyperparameters are optimised by maximising the marginal likelihood of the data (Rasmussen & Williams, 2006). The choice of covariance kernel in effect specifies a model for the data generating process; as described below, using the exponential kernel is equivalent to fitting an Ornstein–Uhlenbeck (OU) process (Uhlenbeck & Ornstein, 1930) to the data.

Standard (stationary) GPs are powerful machine learning methods known for their predictive ability; however, stationary GPs lack the flexibility to model data when there is high function variability in the input space. In an animal movement context, that would correspond to varying characteristics of the trajectories due to different behaviours, such as foraging, hibernation or migration, adopted by the animal. More flexible GP regression can be achieved by allowing the parameters of the covariance kernel and/or the observation noise to vary over space or time (Gibbs, 1997; Paciorek & Schervish, 2004). Heinonen et al. (2016) model the parameters of a non‐stationary covariance kernel with a multi‐layer GP, using Hamiltonian Monte Carlo sampling to sample from the low level GPs defining the kernel parameters. This approach was applied to animal movement data by Torney et al. (2021) to learn time‐varying movement parameters with periodic (seasonal and diurnal) structure. Here, we extend this approach to learning random spatial fields that define the characteristics of a velocity model through the development of a non‐stationary covariance matrix.

### A covariance matrix for non‐stationary correlated velocity models

The correlated random walk (CRW) model of animal movement can be formulated in discrete‐time (McClintock et al., 2012) and continuous‐time (Gurarie, Fleming, et al. 2017). The continuous‐time version employs a correlated velocity model, also called an OU velocity model or integrated OU model. Given that we are dealing with non‐stationary data we wish to derive a non‐stationary version of the correlated velocity model, that is, we wish to derive a covariance matrix that represents the correlation structure in positional observations of an animal following an autocorrelated continuous‐time random walk with varying parameters. Our starting point is therefore an assumed movement model for the animal that is a non‐stationary OU velocity model described by the following equations:

(4) $$\begin{array}{l} df=v\mathrm{dt},\\ dv=-a\left(t\right)v\mathrm{dt}+b\left(t\right)dw_{t}, \end{array}$$

where f is the true location of the animal, v is its velocity, $w_{t}$ is a Wiener process, and $a\left(t\right)$ and $b\left(t\right)$ are time‐varying coefficients that determine the mean‐reversion rate and volatility of the OU process, respectively.

While our movement model is a two‐dimensional model, we will present the derivation of the covariance matrix in the one‐dimensional case to simplify notation and calculations. In the case of constant parameters of the movement process, that is, $a\left(t\right)=a$ and $b\left(t\right)=b$, the covariance function of the OU process is well‐known (Gardiner, 2009) and is equivalent to the exponential covariance function after relaxation of transients terms,

(5) $$\mathrm{Cov}\left(v_{t},v_{s}\right)=\frac{b^{2}}{2a}\exp\left(-a|t-s|\right).$$

To relate the covariance of the velocity process to the covariance of the positions, we note that for a zero‐mean position process

(6) $$\mathrm{Cov}\left(f_{t},f_{s}\right)=E\left(f_{t}f_{s}\right)=E\left(\int_{0}^{t}v_{u}\mathrm{du}\int_{0}^{s}v_{r}\mathrm{dr}\right).$$

(The zero‐mean assumption can always be satisfied by a change of coordinates so that the initial location is at the origin). Through changing the order of integration and application of Fubini's theorem, Equation 6 leads to

(7) $$\mathrm{Cov}\left(f_{t},f_{s}\right)=\int_{0}^{t}\int_{0}^{s}\mathrm{Cov}\left(v_{u},v_{r}\right)\text{dudr}.$$

Hence, the covariance of the position process can be found by performing the double integration of the covariance of the velocity process. For constant parameters of the velocity process, the covariance function defined by Equation 5 may be substituted into Equation 7 and the integral is tractable.

In this work, we are interested in time‐varying velocity characteristics and we therefore employ a non‐stationary version of Equation 5 proposed in Paciorek and Schervish (2004) as

(8) $$\mathrm{Cov}\left(v_{t},v_{s}\right)=\sigma_{\mathrm{st}}^{2}\exp\left(-\frac{|t-s|}{\tau_{\mathrm{st}}}\right),$$

where

(9) $$\begin{array}{l} \sigma_{\mathrm{st}}^{2}=\sigma \left(s\right)\sigma \left(t\right)\sqrt{\frac{2\tau \left(s\right)\tau \left(t\right)}{\tau {\left(s\right)}^{2}+\tau {\left(t\right)}^{2}}},\\ \tau_{\mathrm{st}}=\sqrt{\frac{\tau {\left(s\right)}^{2}+\tau {\left(t\right)}^{2}}{2}}, \end{array}$$

and $\tau \left(t\right),\sigma \left(t\right)$ are the values at time t of the time‐varying kernel timescale and amplitude parameters, respectively. Note that the parameterisation used here differs from that of Equation 5, but there is a direct correspondence between the two.

Substituting Equation 8 into Equation 7 gives

(10) $$\mathrm{Cov}\left(f_{t},f_{s}\right)=\int_{0}^{t}\int_{0}^{s}\sigma_{\mathrm{ru}}^{2}\exp\left(-\frac{|r-u|}{\tau_{\mathrm{ru}}}\right)\text{dudr},$$

which contains an intractable integral due to the non‐constant nature of τ and σ. To approximate a numerical solution to the double integral, we make the following assumption. As we have observations of the animal trajectory at discrete, known time points, we assume that between two successive observations the parameters of the movement process are constant. This assumption means that if we have n observations, the non‐stationary OU process will be split into n−1 piecewise OU processes, with each process having constant parameters. The advantage of this approach is that we can break down the integrals of Equation 7 into segments corresponding to the intervals between observations. Each segment has constant τ and σ values; therefore, the integral can be solved. We then sum over segments to obtain the full integral.

In more detail, given observations at discrete time points $t_{1}$, $t_{2}$, $t_{n}$, where n is the total number of observations, we have

(11) $$\mathrm{Cov}\left(f_{i},f_{j}\right)=\int_{t_{1}}^{t_{i}}\int_{t_{1}}^{t_{j}}\sigma_{\mathrm{ru}}^{2}\exp\left(-\frac{|r-u|}{\tau_{\mathrm{ru}}}\right)\text{dudr}.$$

The inner integral can be written as a sum of integrals with limits corresponding to observation times,

(12) $$\int_{t_{1}}^{t_{2}}\sigma_{\mathrm{ru}}^{2}\exp\left(-\frac{|r-u|}{\tau_{\mathrm{ru}}}\right)\mathrm{du}+\int_{t_{2}}^{t_{3}}\sigma_{\mathrm{ru}}^{2}\exp\left(-\frac{|r-u|}{\tau_{\mathrm{ru}}}\right)\mathrm{du}\ldots +\int_{t_{j-1}}^{t_{j}}\sigma_{\mathrm{ru}}^{2}\exp\left(-\frac{|r-u|}{\tau_{\mathrm{ru}}}\right)\mathrm{du},$$

with a similar decomposition employed for the outer integral. Combined this leads to

(13) $$\mathrm{Cov}\left(f_{i},f_{j}\right)=\sum_{q=1}^{i-1}\sum_{p=1}^{j-1}\int_{t_{q}}^{t_{q+1}}\int_{t_{p}}^{t_{p+1}}\sigma_{\mathrm{ru}}^{2}\exp\left(-\frac{|r-u|}{\tau_{\mathrm{ru}}}\right)\text{dudr}.$$

As each term of the summation corresponds to a pair of between‐observation intervals $\left(p,q\right)$, the parameters of the movement process are taken to be constant. In our model, the movement parameters depend on spatial location; hence, we are unable to specify the true variable values between fixes. In order to specify the true spatially varying value, we would require knowledge of the animal's location between fixes and this information is unavailable due to the discrete nature of the observation process. We therefore must introduce an approximation and we do so by assuming the movement parameters are well approximated by the parameter values at the midpoint of the straight line between the two successive fixes. This gives values $\tau_{p}$, $\sigma_{p}$ and $\tau_{q}$, $\sigma_{q}$ that correspond to the constant parameter values associated with movement in the between‐observation intervals p and q_,_ respectively.

To obtain the parameters required for the non‐stationary covariance kernel, we combine the constant parameters within the observation intervals with Equation 9 to define,

(14) $$\sigma_{\mathrm{pq}}^{2}=\sigma_{p}\sigma_{q}\sqrt{\frac{2\tau_{p}\tau_{q}}{\tau_{p}^{2}+\tau_{q}^{2}}},$$

and

(15) $$\tau_{\mathrm{pq}}=\sqrt{\frac{\tau_{p}^{2}+\tau_{q}^{2}}{2}}.$$

Finally, we end up with a covariance function defined as a summation over a sequence of tractable integrals,

(16) $$\mathrm{Cov}\left(f_{i},f_{j}\right)=\sum_{q=1}^{i-1}\sum_{p=1}^{j-1}\int_{t_{q}}^{t_{q+1}}\int_{t_{p}}^{t_{p+1}}\sigma_{\mathrm{pq}}^{2}\exp\left(-\frac{|r-u|}{\tau_{\mathrm{pq}}}\right)\text{dudr},$$

where the parameters $\sigma_{\mathrm{pq}}$ and $\tau_{\mathrm{pq}}$ are constant within the limits of integration. After some further algebra (see Supplementary Materials for details), the covariance matrix of the non‐stationary integrated OU process for the positions is

(17) $$\begin{array}{ll} \mathrm{Cov}\left(f_{i},f_{j}\right) & =\sum_{q=0}^{i-1}\sum_{p=0}^{j-1}\sigma_{\mathrm{pq}}^{2}\tau_{\mathrm{pq}}^{2}[2,\delta_{\mathrm{pq}},\frac{\left(t_{p+1}-t_{q}\right)}{\tau_{\mathrm{pq}}} \\ & +\exp\left(-\frac{|t_{p}-t_{q+1}|}{\tau_{\mathrm{pq}}}\right)-\exp\left(-\frac{|t_{p+1}-t_{q+1}|}{\tau_{\mathrm{pq}}}\right)\\ & -,\exp,\left(-\frac{|t_{p}-t_{q}|}{\tau_{\mathrm{pq}}}\right),+,\exp,\left(-\frac{|t_{p+1}-t_{q}|}{\tau_{\mathrm{pq}}}\right)], \end{array}$$

where $\delta_{\mathrm{pq}}$ is the Kronecker delta, that is, $\delta_{\mathrm{pq}}=1$, when p=q, and 0 otherwise.

#### Model formulation

In this work, we develop a two‐layer hierarchical GP model, using the non‐stationary integrated OU kernel matrix derived above. In this formulation, the timescale parameter (corresponding to directional persistence) and the variance parameter (corresponding to speed) are modelled by GPs.

The timescale and variance parameters are assumed to depend on the spatial location x (a 2‐d matrix composed of latitude and longitude coordinates), while our observations consist of a vector y that is an n×2 matrix of locations at times t. We assume a regression model for the top layer of our GP hierarchy as,

(18) $$y=f\left(t\right)+\varepsilon ,$$

where $\varepsilon ∼N\left(0,\omega^{2}I\right)$ is a random observation noise term that follows a normal distribution with variance $\omega^{2}$ and I is an $n^{2}\times 2$ identity matrix; hence, we neglect any spatial variation in the error rates of the telemetry equipment and assume a spatially homogeneous measurement error variance. The latent function f corresponds to the (unknown) true location of the animal and we place a GP prior on this vector‐valued function,

(19) $$f\left(t\right)∼\mathrm{GP}\left(y_{0},k_{\mathrm{NS}}\left(t,t^{\prime }\right)\right),$$

where $y_{0}$ is the location of the animal at the first time point and $k_{\mathrm{NS}}\left(t,t^{\prime }\right)$ is the integrated non‐stationary kernel defined by Equation 17. We refer to this as the top layer of our hierarchy and this corresponds to assuming that the animal is following an unbiased CRW (Johnson et al., 2008) with varying characteristics.

Implicit in the specification of the GP prior is the dependence of $k_{\mathrm{NS}}\left(t,t^{\prime }\right)$ on two lower‐level GPs. This is the lower layer of our hierarchy. The log of the timescale (τ) and variance ($\sigma^{2}$) parameters are modelled as latent functions of space and we place separate GP priors on these functions,

(20) $$\begin{array}{l} \tilde{\tau }\left(x\right)∼\mathrm{GP}\left(\mu_{\tau },k_{\tau }\left(x,x^{\prime }\right)\right),\\ {\tilde{\sigma }}^{2}\left(x\right)∼\mathrm{GP}\left(\mu_{\sigma },k_{\sigma }\left(x,x^{\prime }\right)\right). \end{array}$$

To link the layers, we first pass the latent functions through an exponential transform to ensure positivity, and then we translate the spatial dependence of the latent functions to the temporal dependence of the non‐stationary kernel by using the animal's recorded location at time t, that is

(21) $$\begin{array}{l} \tau \left(t\right)=\exp\left[\tilde{\tau }\left(y\left(t\right)\right)\right],\\ \sigma^{2}\left(t\right)=\exp\left[{\tilde{\sigma }}^{2}\left(y\left(t\right)\right)\right]. \end{array}$$

These values enter the top layer GP via Equation 17 of the non‐stationary kernel definition. In order to introduce the effect of environmental features into the model, we may modify Equation 20 so that the mean of the GP depends on an environmental covariate, that is

(22) $$\begin{array}{l} \tilde{\tau }\left(x\right)∼\mathrm{GP}\left(\mu_{L}+\beta_{\tau }.e,k_{\tau }\left(x,x^{\prime }\right)\right),\\ {\tilde{\sigma }}^{2}\left(x\right)∼\mathrm{GP}\left(\mu_{\sigma }+\beta_{\sigma }.e,k_{\sigma }\left(x,x^{\prime }\right)\right), \end{array}$$

where e is a vector of covariate values at the location x_,_ while $\beta_{\tau }$ and $\beta_{\sigma }$ are vectors of coefficients associated with the mean directional persistence and mean speed, respectively.

To complete the model formulation, it remains to specify the covariance kernels of the lower level GPs, $k_{\tau }$ and $k_{\sigma }$. These kernels control the covariance structure of the latent spatial fields and we employ a standard radial basis function kernel (Rasmussen & Williams, 2006) for the empirical data study and a periodic kernel for the synthetic data. This latter choice is dictated by the periodic boundaries of the simulations (see below for details) and would not be an appropriate choice for empirical data.

The log marginal likelihood of a trajectory can then be found by marginalising the probability of the data (the top layer of the GP hierarchy) over the latent functions (the lower layer of the GP hierarchy),

(23) $$\log p\left(y\right)=\log∭p\left(y|f\right)p\left(f|\tau ,\sigma \right)p\left(\tau ,\sigma \right)df\text{dτdσ},$$

where τ and σ are the timescale and variance parameters, respectively, at the observation locations. As the latent functions determine the covariance matrix of the top layer GP, rather than its mean, the integral in Equation 23 is intractable. However, as we show below and in the Supplementary Materials, methods developed for sparse variational GP inference (Hensman et al., 2013; Titsias, 2009) can be applied to the non‐stationary GP model, thereby enabling efficient computation of an approximation to the posterior distribution of the latent functions given the location observations.

#### Model inference

To fit the model to data, we implement our framework using TensorFlow Probability, a probabilistic programming library that is built on TensorFlow, an open‐source deep learning platform (Abadi et al., 2016). In general, GP regression does not scale well with data set size due to the $O\left(N^{3}\right)$ complexity associated with the inversion of the covariance matrix. To compensate for this issue, we employ variational inference (VI) (Blei et al., 2017) in place of MCMC‐sampling. VI proceeds by first proposing a distribution with which to approximate an unknown posterior. Once selected the parameters of the proposed distribution are optimised in order to minimise the distance (typically the Kullback–Leibler divergence) between the approximate distribution and the true posterior. In essence, VI recasts the problem of Bayesian inference as an optimisation problem whereby the parameters of the variational distribution and any hyperparameters associated with the model can be optimised with a gradient descent algorithm (Hoffman et al., 2013).

Details of the inference scheme can be found in the Supplementary Material; however, we describe here two key properties of our approach that ensure the framework is able to run efficiently with large numbers of observations. Firstly, we approximate the full likelihood of a trajectory using a segmentation technique, where we segment individual trajectories into smaller, equal‐sized and more computationally manageable sections. This approach extends the assumption that each GPS collar provides a trajectory that is conditionally independent of others given the latent spatial fields by further breaking trajectories from the same individual into multiple segments. For example, given a trajectory consisting of 4000 observations spanning 2 years, we break this trajectory into 8 segments of 500 observations each spanning a 3‐month period. This method, also known as a mixture of GP experts (Rasmussen & Ghahramani, 2002), has been applied successfully to movement data (Torney et al., 2021) and provides an accurate approximation to the true likelihood if the length of the trajectory segment is large compared to the autocorrelation length of the GP (Snelson & Ghahramani, 2007). In the context of animal movement, this corresponds to selecting trajectory segments with a length greater than the maximum timescale over which directional persistence is observed. Secondly, rather than learning a latent spatial field value for each location of a GPS fix, we define a grid of locations within a fixed domain at which we define the function values for the lower level GPs. To obtain the values of the timescale and variance at the location of an animal (required for Equation 21) we compute the conditional probabilities of the function values at that location given the grid of latent values. This approach reduces the number of latent function values we need to infer and further provides a method for these values to be shared across trajectory segments.

#### Empirical data collection

**FIGURE 1:**
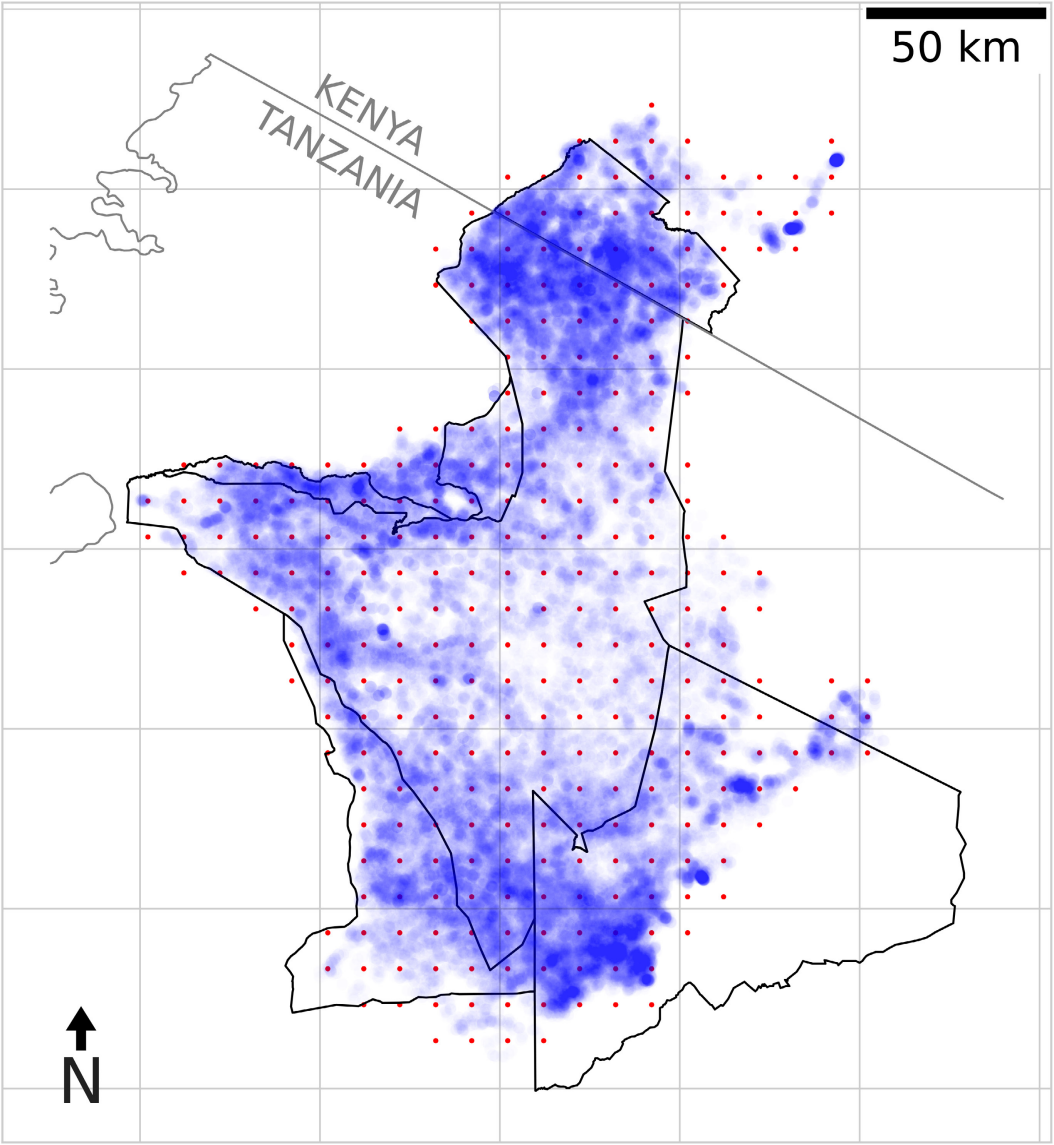
Telemetry locations and inference grid. A map of the Serengeti National Park with GPS locations shown as blue points. The red dots show the inducing grid used for inference of the latent spatial fields.

GPS collars were deployed on 31 migratory wildebeest (*Connochaetes taurinus*) in Serengeti National park, Tanzania, providing a total of 84,000 GPS observations obtained between June 2013 and June 2019. Figure 1 shows a map of the Serengeti National Park along with the recorded locations of wildebeest. The grid of latent function locations used for inference is also shown on the map. In order to explore the movement response of wildebeest to spatially varying features in the landscape, we included metrics associated with both resources and risks as covariates in the non‐stationary GP. Specifically, we used Normalised Difference Vegetation Index (NDVI), grass nitrogen and the distance to drainage beds as the environmental features that might help to explain the observed variation in the directional persistence and speed of wildebeest across the ecosystem. NDVI is a metric of the relative greenness of the vegetation and is associated with quality and productivity of the forage (Boone et al., 2006). The concentration of nitrogen in forage is a proxy of the protein content and is a key component of ungulate diet (Rysava et al., 2016). We also estimated the exposure to risk of natural predation by measuring the animals' proximity to ephemeral drainage lines. In semi‐arid savannas, drainages remain dry for the majority of the year but are associated with landscape features such as thick vegetation, river confluences, and erosion terraces, all of which conceal ambush predators such as lions and improve their success of catching prey (Davidson et al., 2012; Hopcraft et al., 2005). Previous studies have shown that ungulates reduce the risk of predation by avoiding these areas (Anderson et al., 2010; Hopcraft et al., 2012, 2014). Further details on data collection can be found in the Supplementary Materials.

#### Synthetic data generation

For the generation of the synthetic data set, we simulate from a non‐stationary CRW model, where the parameters of the velocity process, mean‐reversion, a, and the volatility of the OU process, b are position‐dependent,

(24) $$\begin{array}{l} df=v\mathrm{dt},\\ dv=-a\left(f\right)v\mathrm{dt}+b\left(f\right)dw_{t}. \end{array}$$

**FIGURE 2:**
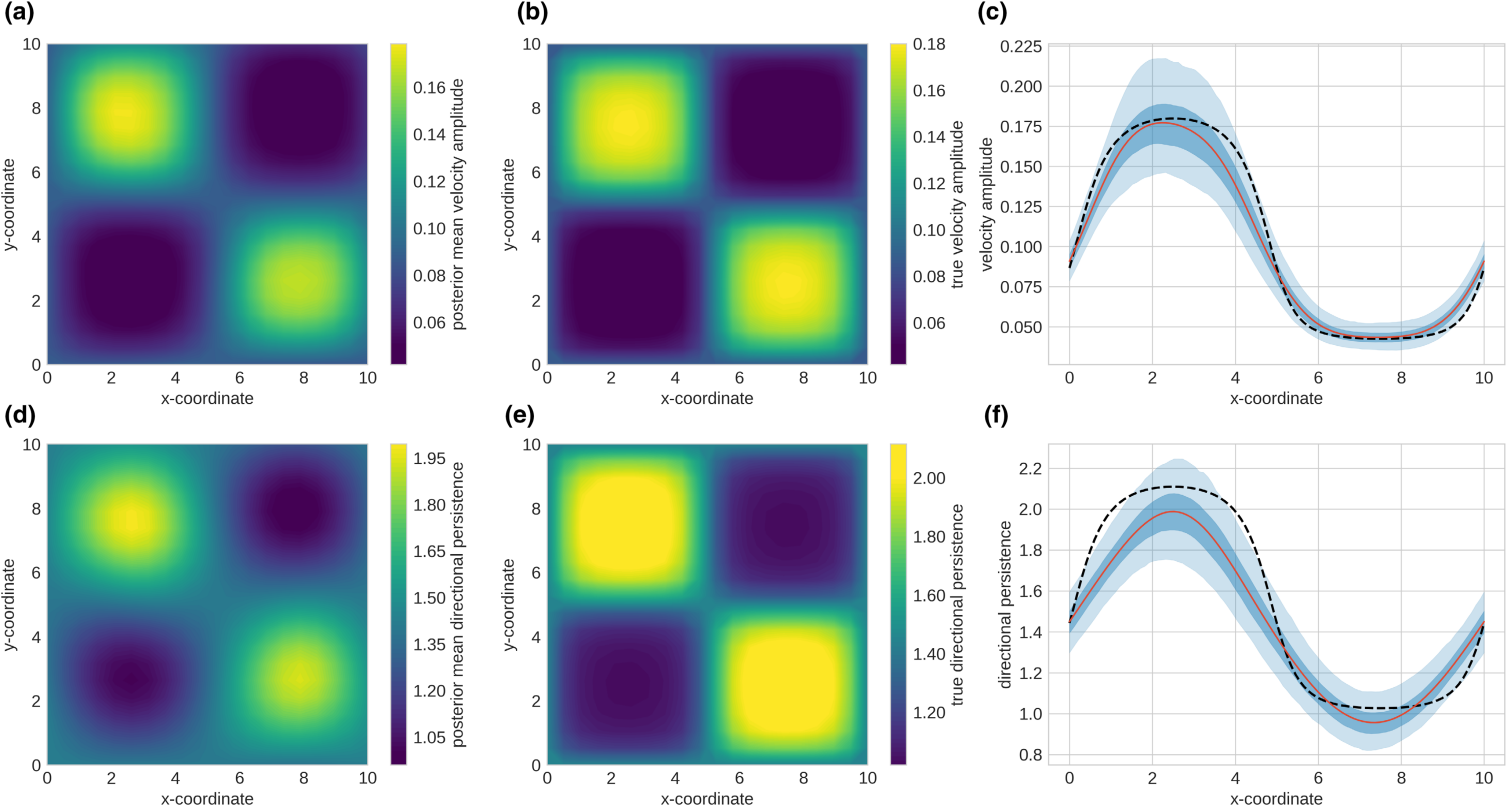
Simulation model inference. (a) and (b) show the inferred kernel variance and approximate ground truth value of the kernel variance, respectively. (d) and (e) show the inferred timescale and the approximate ground truth kernel timescale, respectively. (c) and (f) show a one‐dimensional profile with uncertainty; the black dashed line is the approximate ground truth value of the parameters, the red line is the posterior mean and the dark blue region is the 50% CI and the light blue region is the 90% CI.

The movement process gives rise to positional observations of the animal at discrete time points that are subject to observation error, so that y=f+ε where ε is a white noise term. We create the spatial fields for $a\left(f\right)$ and $b\left(f\right)$ using a two‐dimensional version of the warped sine function,

(25) $$\text{wsin}\left(v\right)=\sqrt{\frac{1+\alpha^{2}}{1+\alpha^{2}\sin^{2}\left(2\mathrm{\pi v}\right)}}\sin\left(2\mathrm{\pi v}\right),$$

where α=2 gives a flattened sine wave that provides a more patch‐like environment. The spatial field used to generate the movement trajectories is shown in Figure 2.

To account for the finite simulation domain, we introduce periodic boundary conditions for the environment. This creates an infinite domain on which the simulated animals move, but they encounter a repeating, tiled spatial field if they cross the boundaries of the environment. In this way the boundaries of the simulation do not affect the movement of the individuals and all spatially varying movement characteristics result from the latent fields. We simulate 200 individuals moving across an environment and collect 500 positional observations from each individual resulting in 100,000 observations. To measure the execution time of our model, we also generate synthetic data sets ranging from 100,000 observations to 1.6 million observations.

## RESULTS

To fit the hierarchical model to data and infer the latent spatial fields, we maximise a lower bound on the log‐marginal likelihood (see Supplementary Material) which is equivalent to minimising the Kullback–Leibler divergence between the variational distribution and the true posterior distribution. We use stochastic gradient descent and the Adam optimiser (Kingma & Ba, 2017) to achieve this with the negative of the lower bound acting as the loss function to be minimised.

### Simulation model

We ran the optimiser and monitored the loss over time to assess convergence. Training was automatically halted if the loss had not decreased over 5 epochs (one epoch corresponded to one iteration through the entire data set). Training loss is shown in Fig S1. The mean of the posterior distributions of the latent spatial fields is shown in Figure 2. As we are using simulated data, this can be compared to the values used to create the movement trajectories. We observe a very close agreement between the inferred values and the simulated environment. We note that there is not an exact match between the hierarchical GP model and the simulation model; however, we are able to accurately locate the regions of different movement characteristics and recover the parameter values within the regions. The model is unable to perfectly capture the shape of the timescale function as it transitions between regions and this is due to the transient dynamics in the velocity process when an animal enters a region where its directional persistence alters. When the degree of persistence alters there is a delay before this is detectable in the data, and hence a blurring of the borders between regions.

To visualise the uncertainty, we show one‐dimensional profiles of the true environment, inferred values and credible intervals in Figure 2c,f. We further investigate the execution time of our algorithm and show that we are able to process data sets of 1.6 million observations in under 3.5 h (see Fig. S2).

#### Wildebeest movement

Inferring the spatial characteristics of the wildebeest migration followed a similar approach to the synthetic data. Again we ran the optimiser until the model had converged, that is the loss had not decreased for 5 epochs (see Figs. S3 and S4). Training took around 22 min for 84,000 observations on an NVIDIA Quadro GP100 GPU. Full movement trajectories of the wildebeest were split into trajectory segments consisting of 500 points which equated to roughly 3 months depending on the sampling schedule of the collar. We employed a batch size of 4, meaning 4 trajectory segments were passed into the optimiser at each iteration, with the data set shuffled at the end of each epoch.

**FIGURE 3:**
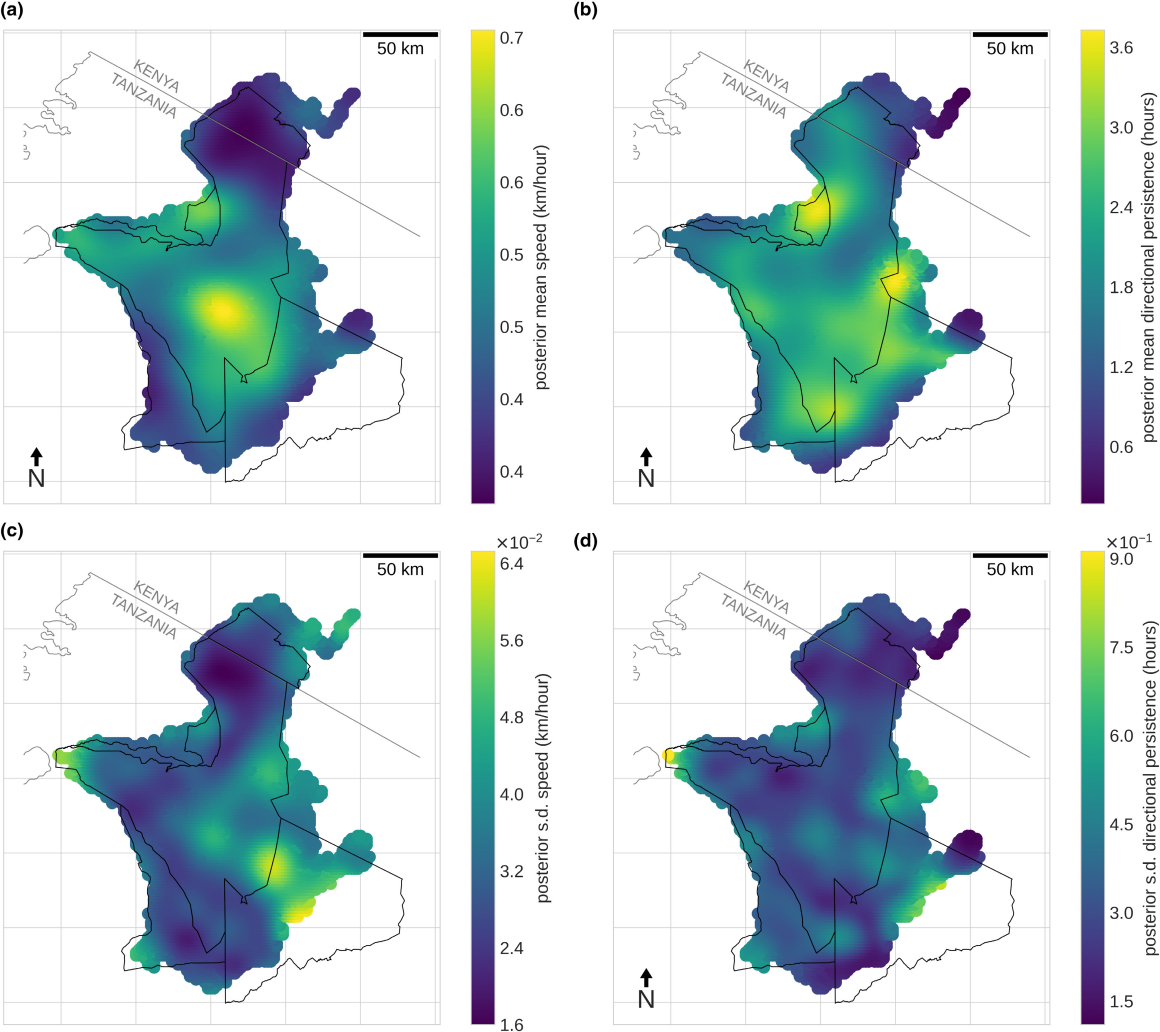
Empirical data inference. (a) Posterior mean speed calculated from the variational posterior distribution. (b) Posterior mean directional persistence. (c) Speed standard deviation of variational posterior distribution. (d) Directional persistence standard deviation of variational posterior distribution.

Inferred posterior means for the latent fields are shown in Figure 3. For uncertainty quantification, we also show the posterior standard deviation of the field, along with the 95% credible intervals for the posterior samples in Figure S5. Our results reveal the migratory pathways of the wildebeest; regions of high directional persistence can be found in a circuit around the southern extent of the Serengeti, corresponding to the main pathway that moves south along the east of the park and then north through the western corridor. A region of high speeds, but low directional persistence, can be found at the centre of the migration where the long grass plains of the Serengeti are found. We expect that this pattern can be attributed to rapid transit between the nutrient‐rich short grass plains in the south‐east and the woodland areas of the Western corridor.

We further detect significantly different movement behaviour in the north west of the park close to the boundary and south of the Tanzania‐Kenya border. Here, we observe high speeds and high directional persistence, meaning we can identify a region through which wildebeest move directly and rapidly. This is a region of high human density and, while we can not attribute causality, our results are strongly suggestive of an effect of human presence on the movement behaviour of wildebeest (Rija & Kideghesho, 2020). Finally, we note that uncertainty in the spatial fields is in general low. High uncertainty is only found at the edges of the wildebeest's migratory range in regions of very little data. This highlights a key advantage of our Bayesian approach. We observe higher speeds at the centre of the park and in the north‐west close to villages and human activity. Since these locations have low uncertainty, we can be confident that we are detecting regions of significantly different movement behaviour.

**FIGURE 4:**
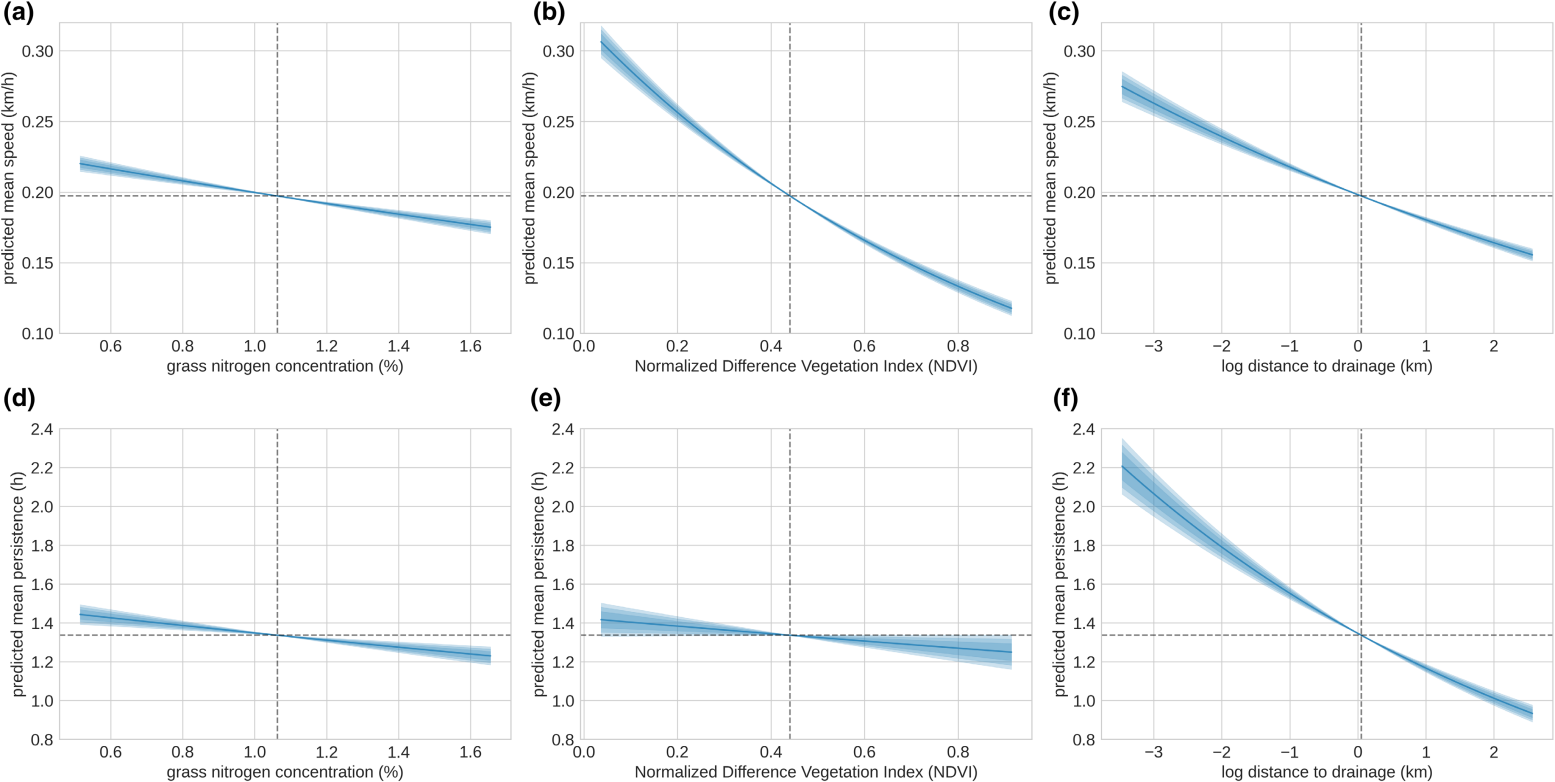
Effect of resources and risk on movement. (a–c) Mean speed as a function of covariate. (d–f) Mean directional persistence as a function of covariate. Horizontal dashed line indicates the model intercept, and vertical dashed line indicates the mean covariate value in the data set.

We next extend the model to include the effects of the environmental covariates, grass nitrogen, NDVI and distance to drainage, on the mean values of the movement parameters. Results from this analysis are shown in Figure 4. In line with previous studies (Stabach et al., 2022), we find that NDVI values have a significant effect on the average speed of wildebeest, with lower speeds being associated with high quality forage as would be expected. A weaker effect of grass nitrogen on movement speed was found. This likely indicates that although nitrogen is associated with grass quality and protein content, wildebeest are strongly influenced by instantaneous conditions and the ephemeral nature of resources in the Serengeti (Holdo et al., 2009) makes NDVI a better predictor of movement patterns. Grass nitrogen did have a small effect on directional persistence, with less directed movements associated with regions of high grass quality. This could be caused by irregular movements of animals tracking ephemeral resources in the wet season, as observed by Hopcraft et al. (2014). Increased distance to drainage, which we associate with lower predation risk, had a greater effect on directional persistence, along with a large effect on movement speeds. These results indicate that wildebeest make significantly faster, more directed movements through regions associated with high predation risk (i.e. regions close to drainage lines). An interesting extension to this analysis would be to compare this observed response to natural predation with the response to human‐caused mortality risk.

## DISCUSSION

In this work, we present a Bayesian hierarchical framework for learning the latent spatial fields that underlie observed animal movement patterns. Our framework links two fundamental concepts in statistical ecology: spatial random fields and CRW models of animal movement. As both these methods can be formulated as GPs, we adopt a multi‐layer GP approach implemented within the high‐performance machine learning package, TensorFlow. Our framework has several advantages over existing approaches to animal movement modelling. Multi‐layer GPs offer a flexible, non‐parametric method of inferring latent spatial fields as well as providing an effective means to model spatially correlated residuals when fitting parametric models. However, while we are not required to make any restrictive assumptions about the functional form of the underlying field, we can encode prior knowledge into the kernel functions of the low level GPs by employing appropriate covariance kernels.

In order to efficiently process large data sets, we employ stochastic VI and a trajectory segmentation technique that circumvents the cubic scaling of GPs with data set size (Rasmussen & Williams, 2006). While segmentation does introduce artificial breakpoints into continuous trajectories, this will not affect the model inference as long as segments are long compared to the timescale over which an animal's velocity is autocorrelated. For practical applications, this means that location fix rates should be set, or subsampled, so that a segment spans a time period that exceeds the typical velocity autocorrelation time.

As animal movement is inherently a multiscale process (Torney, Hopcraft, et al. 2018) in which animals respond to multiple, often contradictory cues (Hopcraft et al., 2014), a latent spatial field approach can offer key insights into the different behaviours that animals exhibit across a landscape, enabling specific regions to be associated with certain behaviours. This can be achieved without having to make decisions about which environmental features to include in a model, or how to discretise movement data into the individual choices of an animal, and can provide information on migratory routes, foraging grounds or regions of high perceived risk. While our model can infer latent spatial fields based on location data alone, it can also incorporate specific covariates if required. Hence, the parameters of the movement model can be associated with environmental features to explore the drivers of observed spatial patterns and the response of animals to resources and risks in the landscape.

Since our method learns spatial patterns of movement directly from data and by‐passes the need for hypothesised relationships to environmental covariates, it creates opportunities in several ecological application areas. Notably, our approach enables the investigation of human‐mediated environmental disturbance on animal movement, even when disturbance effects are non‐local (Kavwele et al., 2022) or the level of human activity is difficult to measure. By detecting regions of markedly different movement behaviours, our analysis is able to act as a precursor to environmental data collection rather than being dependent on the prior identification and investigation of spatial covariates. Hence, our approach is able to direct data collection efforts to regions where movement behaviour is significantly different as compared to other locations or recent history. Finally, our method is able to process large‐‐scale data sets consisting of location observations from multiple individuals by leveraging techniques from the domain of Bayesian machine learning (Ghahramani, 2015). As the resolution and volume of animal movement data increase, it seems likely that many of these techniques will become invaluable tools for ecologists seeking to extract meaningful insight from this wealth of telemetry data (Nathan et al., 2022).

## AUTHOR CONTRIBUTION

CJT, DH, IP and GH designed the study, IP, CJT and MMM performed the analysis, GH collected the data and CJT, IP, DH and GH wrote the manuscript.

### PEER REVIEW

The peer review history for this article is available at https://publons.com/publon/10.1111/ele.14117.

## Supporting information


Appendix S1.

## Data Availability

The code used to generate all figures and analysis is freely available at https://doi.org/10.5281/zenodo.7079680. Data used in the study are available here: https://doi.org/10.5061/dryad.5tb2rbp76
