# Supplementary material for: Inferring spatially varying animal movement characteristics using a hierarchical continuous‐time velocity model
Source: Ecol Lett. 2022 Oct 18;25(12):2726–38. doi: 10.1111/ele.14117 (PMC9828272; doi:10.1111/ele.14117)
Supplement: Supplementary file 1 — Appendix S1. [file ELE-25-2726-s001.pdf]

SUPPLEMENTAL MATERIALS

1 | DERIVATION OF THE INTEGRATED NON-STATIONARY OU COVARIANCE FUNCTION

From the main text, we have that the integrated non-stationary OU covariance function has the following form

$$\operatorname{Cov}\left(f_i, f_j\right) = \sum_{q=1}^{i-1} \sum_{p=1}^{j-1} \int_{t_q}^{t_{q+1}} \int_{t_p}^{t_{p+1}} \sigma_{ru}^2 \exp\left(-\frac{|r-u|}{\tau_{ru}}\right) du dr. \tag{S1}$$

To solve this equation, we now consider three cases: $p = q$, $p > q$ and $p < q$. This is done due to the modulus inside the double integral, as we need to consider the relationship between u and r, and consequently the relationship between p and q. Due to symmetry, the latter two cases will be analogous. We denote the double integral term inside Eq. S1 as I for simplicity.

Firstly, if $p = q$, then we have within the integration interval a region where $u < r$ and a region where $u > r$. To account for the modulus term $|r - u|$, we split the inner integral into two integrals with appropriate integral bounds such that the modulus disappears. We then calculate the integrals individually. This gives,

$$\begin{aligned}
I &= \sigma_{pp}^2 \int_{t_p}^{t_{p+1}} \int_{t_p}^{t_{p+1}} \exp\left(-\frac{|u-r|}{\tau_{pp}}\right) du dr = \sigma_{pp}^2 \int_{t_p}^{t_{p+1}} \left[\int_{t_p}^{r} \exp\left(-\frac{(r-u)}{\tau_{pp}}\right) du + \int_{r}^{t_{p+1}} \exp\left(-\frac{(u-r)}{\tau_{pp}}\right) du \right] dr \\
&= \sigma_{pp}^2 \int_{t_p}^{t_{p+1}} \left[\tau_{pp} \exp\left(-\frac{(r-u)}{\tau_{pp}}\right) \Bigg|_{t_p}^{r} - \tau_{pp} \exp\left(-\frac{(u-r)}{\tau_{pp}}\right) \Bigg|_{r}^{t_{p+1}} \right] dr \\
&= \sigma_{pp}^2 \tau_{pp} \int_{t_p}^{t_{p+1}} \left[1 - \exp\left(-\frac{(r-t_p)}{\tau_{pp}}\right) - \exp\left(-\frac{(t_{p+1}-r)}{\tau_{pp}}\right) + 1 \right] dr \\
&= \sigma_{pp}^2 \tau_{pp} \left[2r + \tau_{pp} \exp\left(-\frac{(r-t_p)}{\tau_{pp}}\right) - \tau_{pp} \exp\left(-\frac{(t_{p+1}-r)}{\tau_{pp}}\right) \right]_{t_p}^{t_{p+1}} \\
&= \sigma_{pp}^2 \tau_{pp} \left[2(t_{p+1}-t_p) + \tau_{pp} \exp\left(-\frac{\left(t_{p+1}-t_p\right)}{\tau_{pp}}\right) - \tau_{pp} - \tau_{pp} + \tau_{pp} \exp\left(-\frac{\left(t_{p+1}-t_p\right)}{\tau_{pp}}\right) \right] \\
&= 2\sigma_{pp}^2 \tau_{pp}^2 \left[\frac{\left(t_{p+1}-t_p\right)}{\tau_{pp}} + \exp\left(-\frac{\left(t_{p+1}-t_p\right)}{\tau_{pp}}\right) - 1 \right].
\end{aligned} \tag{S2}$$

In the second case, when $p > q$, we have that $t_p \geq t_{q+1}$ and hence $u > r$ throughout and the modulus term can be replaced with $u - r$. We calculate each integral in turn to give,

$$\begin{aligned} I &= \sigma_{pq}^2 \int_{t_q}^{t_{q+1}} \int_{t_p}^{t_{p+1}} \exp\left(-\frac{(u-r)}{\tau_{pq}}\right) du dr = \sigma_{pq}^2 \int_{t_q}^{t_{q+1}} \left[-\tau_{pq} \exp\left(-\frac{(u-r)}{\tau_{pq}}\right)\Big|_{t_p}^{t_{p+1}} dr \right] \\ &= \sigma_{pq}^2 \tau_{pq} \int_{t_q}^{t_{q+1}} \exp\left(-\frac{(t_p - r)}{\tau_{pq}}\right) - \exp\left(-\frac{(t_{p+1} - r)}{\tau_{pq}}\right) dr = \sigma_{pq}^2 \tau_{pq}^2 \left[\exp\left(-\frac{(t_p - r)}{\tau_{pq}}\right) - \exp\left(-\frac{(t_{p+1} - r)}{\tau_{pq}}\right) \right]_{t_q}^{t_{q+1}} \\ &= \sigma_{pq}^2 \tau_{pq}^2 \left[\exp\left(-\frac{(t_p - t_{q+1})}{\tau_{pq}}\right) - \exp\left(-\frac{(t_{p+1} - t_{q+1})}{\tau_{pq}}\right) - \exp\left(-\frac{(t_p - t_q)}{\tau_{pq}}\right) + \exp\left(-\frac{(t_{p+1} - t_q)}{\tau_{pq}}\right) \right]. \end{aligned} \tag{S3}$$

Keeping in mind the symmetry in p and q we can rewrite the last equation as

$$I = \sigma_{pq}^2 \tau_{pq}^2 \left[\exp\left(-\frac{|t_p - t_{q+1}|}{\tau_{pq}}\right) - \exp\left(-\frac{|t_{p+1} - t_{q+1}|}{\tau_{pq}}\right) - \exp\left(-\frac{|t_p - t_q|}{\tau_{pq}}\right) + \exp\left(-\frac{|t_{p+1} - t_q|}{\tau_{pq}}\right) \right]. \tag{S4}$$

Therefore, we have that the non-stationary integrated OU covariance function is

$$\begin{aligned} \mathrm{Cov}\left(f_i, f_j\right) = \sum_{q=0}^{i-1} \sum_{p=0}^{j-1} \sigma_{pq}^2 \tau_{pq}^2 \Bigg[2\delta_{pq} \frac{(t_{p+1} - t_q)}{\tau_{pq}} + \exp\left(-\frac{|t_p - t_{q+1}|}{\tau_{pq}}\right) - \exp\left(-\frac{|t_{p+1} - t_{q+1}|}{\tau_{pq}}\right) - \exp\left(-\frac{|t_p - t_q|}{\tau_{pq}}\right) \\ + \exp\left(-\frac{|t_{p+1} - t_q|}{\tau_{pq}}\right) \Bigg], \end{aligned} \tag{S5}$$

where $\sigma_{pq}^2 = \sigma_p \sigma_q \sqrt{\frac{2\tau_p \tau_q}{\tau_p^2 + \tau_q^2}}$, $\tau_{pq} = \sqrt{\frac{\tau_p^2 + \tau_q^2}{2}}$ and $\delta_{pq} = 1$, when $p = q$, and 0 otherwise.

2 | EMPIRICAL DATA COLLECTION

GPS collars (Followit, formerly 'Televilt,' GSM or Iridium transmitters with GPS location) were deployed on 31 migratory wildebeest (*Connochaetes taurinus*) in Serengeti National park, Tanzania. Animals were immobilized by veterinarians from the Tanzania Wildlife Research Institute (TAWIRI) or the Tanzania National Parks (TANAPA) using an injectable dart containing 4-6 mg of etorphine and 80–100 mg of azaperone, fired from a veterinary rifle from a stationary vehicle near the animal. Veterinarians followed the handling and care protocols established by TAWIRI.

Collared animals were healthy reproductively active adult females (>2 years old) that were selected at random with an attempt to ensure collars were distributed throughout the main aggregations of the herds. A total of 84,000 GPS observations were obtained between June 2013 and June 2019. Collars were either collected after 2-3 years of deployment using a remote-release mechanism, or collected in the field after a mortality event. Collars were continually redeployed during the study with the last deployment occurring in March 2018. Animals included in the study were tracked for periods ranging from 183 days to 1119 days.

We used Normalized Difference Vegetation Index (NDVI), grass nitrogen, and the distance to drainage beds as the environmental features that might help to explain the observed variation in the directional persistence and speed of wildebeest across the ecosystem. We used the 16-day NDVI product from the MODIS Terra satellite at a 250m resolution. NDVI values range from 0 (indicating dry conditions with low quality food) to 1 (indicating relatively green conditions with high food abundance). In addition we used, a spatially interpolated metric of grass nitrogen derived from empirical measures of clipped grasses from 148 sites from across the ecosystem (details described in Hopcraft et al. (2012)). At each site five 25x25cm quadrats were clipped, dried, ground to 2mm particle size, and scanned using Near Infrared Absorption Spectrometer to estimate the percent nitrogen in each sample. We created a spatially continuous layer of grass nitrogen by regression kriging the mean nitrogen value at each site with the long-term mean NDVI. The final product was an ecosystem-wide raster estimating the mean percent nitrogen in the grass at a 1km resolution. For every wildebeest GPS location, we extracted the estimated grass nitrogen content and included this as measure of the quality of the resource. We also estimated the exposure to risk of natural predation by measuring the animals' proximity to ephemeral drainage lines.

3 | VARIATIONAL INFERENCE PROCEDURE

Variational inference (VI) is an alternative Bayesian inference method that replaces more traditional sampling based approaches such as MCMC with a more efficient but approximate method for obtaining posterior distributions. In VI we optimise the parameters of a variational distribution that is an approximation to the true posterior distribution. This is achieved by maximising a lower bound for the marginal likelihood, which is equivalent to minimising the Kullback-Leibler divergence between the approximate variational distribution and the exact posterior distribution. VI has been widely used in GP regression as it enables the processing of very large datasets and the use of non-Gaussian likelihoods.

In this paper, we employ VI to obtain posterior distributions for the parameters contained within our model defined by Eqs. 18-20 in the main text. Our methodology is based on existing approaches (Hensman et al. 2013; Salimbeni & Deisenroth 2017; Titsias 2009) in the literature but some modifications are made in order to apply VI to our specific use case. Mainly we must account for the difficulty associated with the latent functions entering the likelihood via the covariance kernel and the extension to two spatial dimensions with shared kernel parameters.

We first define m inducing points at locations $\mathbf{z}$ (a 2-d matrix composed of spatial locations with shape $m \times 2$) with the corresponding latent functions $\boldsymbol{\tau}_z$ and $\boldsymbol{\sigma}_z$ (both latent functions have shape $m \times 1$). At the observation locations $\mathbf{x}$ (a 2-d matrix composed of latitude and longitude coordinates of shape $n \times 2$) the corresponding latent functions are $\boldsymbol{\tau}$ and $\boldsymbol{\sigma}$ (both have shape $n \times 1$). The choice of the number of inducing points and their locations is domain specific but typically m is chosen such that the distance between points is less than the scale over which the landscape changes, $m \ll n$, and the locations are evenly distributed

throughout the domain. Selecting a value of m that is too high will slow down the inference algorithm, while a value that is too low will mean that fine scale features of the landscape will not be captured.

Our goal is to obtain an approximation to the posterior distribution, $p(\boldsymbol{\tau}_z, \boldsymbol{\sigma}_z|\mathbf{y})$, from which we can obtain the posterior of the latent functions at arbitrary locations, $p(\boldsymbol{\tau}^*, \boldsymbol{\sigma}^*|\mathbf{y})$. In order to find this approximate distribution we first propose a form for the distribution, termed the variational distribution, then minimize the Kullback-Leibler divergence between this distribution and the true posterior by maximizing a lower bound for the marginal likelihood. Note in what follows we omit to use the notation $\tilde{\boldsymbol{\tau}}$ and $\tilde{\boldsymbol{\sigma}}$ when referring to the latent functions to aid clarity, however when calculating covariance matrices the values do undergo an exponential transformation to ensure positivity.

To derive the lower bound we first make the assumption that,

$$p(\mathbf{y}|\boldsymbol{\tau}, \boldsymbol{\sigma}, \boldsymbol{\tau}_z, \boldsymbol{\sigma}_z) = p(\mathbf{y}|\boldsymbol{\tau}_z, \boldsymbol{\sigma}_z), \tag{S6}$$

which in effect means that we assume that the latent function values at the inducing locations are sufficient statistics for the function values at all locations. Using this assumption, the posterior distribution of the latent functions at the observation locations is

$$p(\boldsymbol{\tau}, \boldsymbol{\sigma}, \boldsymbol{\tau}_z, \boldsymbol{\sigma}_z|\mathbf{y}) = p(\boldsymbol{\tau}|\boldsymbol{\tau}_z)p(\boldsymbol{\sigma}|\boldsymbol{\sigma}_z)p(\boldsymbol{\tau}_z, \boldsymbol{\sigma}_z|\mathbf{y}), \tag{S7}$$

where $p(\boldsymbol{\tau}_z, \boldsymbol{\sigma}_z|\mathbf{y})$ is the joint posterior distribution at the inducing points locations and $p(\boldsymbol{\tau}|\boldsymbol{\tau}_z)$, $p(\boldsymbol{\sigma}|\boldsymbol{\sigma}_z)$ can be found in closed form by using conditional probabilities of Gaussian distributions and the fact that we have placed a GP prior on the latent function values.

Variational inference is performed by introducing a variational distribution ϕ that approximates the true posterior distribution such that

$$p(\boldsymbol{\tau}, \boldsymbol{\sigma}, \boldsymbol{\tau}_z, \boldsymbol{\sigma}_z|\mathbf{y}) \approx q(\boldsymbol{\tau}, \boldsymbol{\sigma}, \boldsymbol{\tau}_z, \boldsymbol{\sigma}_z) = p(\boldsymbol{\tau}|\boldsymbol{\tau}_z)p(\boldsymbol{\sigma}|\boldsymbol{\sigma}_z)\phi(\boldsymbol{\tau}_z, \boldsymbol{\sigma}_z), \tag{S8}$$

where the variational distribution ϕ has the following form

$$\phi(\boldsymbol{\tau}_z, \boldsymbol{\sigma}_z) \sim \mathcal{N}\left(\boldsymbol{\mu}_q, \mathbf{K}_q\right). \tag{S9}$$

The log marginal likelihood is

$$\log p(\mathbf{y}) = \log \iiiint p(\mathbf{y}|\boldsymbol{\tau}, \boldsymbol{\sigma})p(\boldsymbol{\tau}, \boldsymbol{\sigma}|\boldsymbol{\tau}_z, \boldsymbol{\sigma}_z)p(\boldsymbol{\tau}_z)p(\boldsymbol{\sigma}_z)d\boldsymbol{\tau}d\boldsymbol{\sigma}d\boldsymbol{\tau}_z d\boldsymbol{\sigma}_z. \tag{S10}$$

Before deriving a lower bound on this term, we first define $q(\boldsymbol{\tau}, \boldsymbol{\sigma}) = \iint q(\boldsymbol{\tau}, \boldsymbol{\sigma}, \boldsymbol{\tau}_z, \boldsymbol{\sigma}_z)d\boldsymbol{\tau}_z d\boldsymbol{\sigma}_z$ and introduce the notation $\mathcal{KL}(p||q)$ for the Kullback-Leibler divergence between two distributions p and q,

$$\mathcal{KL}(p||q) = \int p(x) \log\left(\frac{q(x)}{p(x)}\right) dx. \tag{S11}$$

With these definitions, the lower bound for the marginal log-likelihood can be found as,

$$\begin{aligned}
\log p(\mathbf{y}) &= \log \iiiint p(\mathbf{y}|\boldsymbol{\tau},\boldsymbol{\sigma})p(\boldsymbol{\tau}|\boldsymbol{\tau}_z)p(\boldsymbol{\sigma}|\boldsymbol{\sigma}_z)p(\boldsymbol{\tau}_z)p(\boldsymbol{\sigma}_z)d\boldsymbol{\tau}d\boldsymbol{\sigma}d\boldsymbol{\tau}_z d\boldsymbol{\sigma}_z \\
&= \log \iiiint p(\mathbf{y}|\boldsymbol{\tau},\boldsymbol{\sigma})p(\boldsymbol{\tau}|\boldsymbol{\tau}_z)p(\boldsymbol{\sigma}|\boldsymbol{\sigma}_z)p(\boldsymbol{\tau}_z)p(\boldsymbol{\sigma}_z)\frac{q(\boldsymbol{\tau},\boldsymbol{\sigma},\boldsymbol{\tau}_z,\boldsymbol{\sigma}_z)}{q(\boldsymbol{\tau},\boldsymbol{\sigma},\boldsymbol{\tau}_z,\boldsymbol{\sigma}_z)}d\boldsymbol{\tau}d\boldsymbol{\sigma}d\boldsymbol{\tau}_z d\boldsymbol{\sigma}_z \\
&\geq \iiiint \log\left(\frac{p(\mathbf{y}|\boldsymbol{\tau},\boldsymbol{\sigma})p(\boldsymbol{\tau}|\boldsymbol{\tau}_z)p(\boldsymbol{\sigma}|\boldsymbol{\sigma}_z)p(\boldsymbol{\tau}_z)p(\boldsymbol{\sigma}_z)}{q(\boldsymbol{\tau},\boldsymbol{\sigma},\boldsymbol{\tau}_z,\boldsymbol{\sigma}_z)}\right) q(\boldsymbol{\tau},\boldsymbol{\sigma},\boldsymbol{\tau}_z,\boldsymbol{\sigma}_z)d\boldsymbol{\tau}d\boldsymbol{\sigma}d\boldsymbol{\tau}_z d\boldsymbol{\sigma}_z \qquad \text{(S12)} \\
&= \iint \log\left(p(\mathbf{y}|\boldsymbol{\tau},\boldsymbol{\sigma})\right) q(\boldsymbol{\tau},\boldsymbol{\sigma})d\boldsymbol{\tau}d\boldsymbol{\sigma} - \mathcal{KL}(\phi(\boldsymbol{\tau}_z,\boldsymbol{\sigma}_z)||p(\boldsymbol{\tau}_z)p(\boldsymbol{\sigma}_z)) \\
&= \iint \log\left(p(\mathbf{y}_1|\boldsymbol{\tau},\boldsymbol{\sigma})\right) q(\boldsymbol{\tau},\boldsymbol{\sigma})d\boldsymbol{\tau}d\boldsymbol{\sigma} + \iint \log\left(p(\mathbf{y}_2|\boldsymbol{\tau},\boldsymbol{\sigma})\right) q(\boldsymbol{\tau},\boldsymbol{\sigma})d\boldsymbol{\tau}d\boldsymbol{\sigma} - \mathcal{KL}(\phi(\boldsymbol{\tau}_z,\boldsymbol{\sigma}_z)||p(\boldsymbol{\tau}_z)p(\boldsymbol{\sigma}_z)),
\end{aligned}$$

where Jensen's inequality is applied at the first inequality and we have used $\mathbf{y}_1$ and $\mathbf{y}_2$ to refer to the two spatial dimensions of the observations (corresponding to latitude and longitude coordinates) which are conditionally independent given the latent function values.

The $\mathcal{KL}$ divergence term contained in Eqn. S12 is analytically tractable since both distributions are multivariate Normal distributions. Similarly, the term $q(\boldsymbol{\tau},\boldsymbol{\sigma})$ is tractable due to the marginalisation property of the multivariate normal distribution. Finally, given the trajectory segmentation approximation described in the main text, the integral terms in Eqn. S12 can be factorised such that we have

$$\begin{aligned}
\iint \log\left(p(\mathbf{y}_j|\boldsymbol{\tau},\boldsymbol{\sigma})\right)q(\boldsymbol{\tau},\boldsymbol{\sigma})d\boldsymbol{\tau}d\boldsymbol{\sigma} &= \iint \log\left(\prod_{i=1}^{S} p(\mathbf{y}_j^i|\boldsymbol{\tau}_i,\boldsymbol{\sigma}_i)\right) q(\boldsymbol{\tau},\boldsymbol{\sigma})d\boldsymbol{\tau}d\boldsymbol{\sigma} \\
&= \sum_{i=1}^{S} \iint \log\left(p(\mathbf{y}_j^i|\boldsymbol{\tau}_i,\boldsymbol{\sigma}_i)\right)q(\boldsymbol{\tau}_i,\boldsymbol{\sigma}_i)d\boldsymbol{\tau}_i d\boldsymbol{\sigma}_i,
\end{aligned} \qquad \text{(S13)}$$

where S is the number of independent segments we divide the data into, $j \in \{1,2\}$ is the spatial dimension, and $\mathbf{y}_j^i$ indicates the ith segment in dimension j.

To calculate the expected log-likelihood term we use the Monte Carlo sampling method (Salimbeni & Deisenroth 2017). More specifically, we draw 32 samples from the multivariate Normal distribution $q(\boldsymbol{\tau}_i,\boldsymbol{\sigma}_i)$ then, for each sample we draw we calculate the log-likelihood of a trajectory segment, $\log p(\mathbf{y}^i{}_j|\boldsymbol{\tau}_i^k,\boldsymbol{\sigma}_i^k)$, where $\boldsymbol{\tau}_i^k$ is the kth sample for the ith segment. Finally, we average over all the samples in order to calculate the expected log likelihood term. Hence, the lower bound can be maximised using stochastic optimisation and the optimal parameters of the variational distribution can be found.

To improve numerical performance, we use Cholesky whitening for the variational parameters such that $\boldsymbol{\phi} = \boldsymbol{\mu}_q + \mathbf{L}\mathbf{v}$, where $\mathbf{L}\mathbf{L}^T = \mathbf{K}_q$ and $\mathbf{v} \sim \mathcal{N}(\mathbf{0},\mathbf{I})$. We then optimise all parameters including the the variational parameters $\boldsymbol{\mu}_q$, the whitened variables $\mathbf{v}$, all hyperparameters, and the locations of the inducing points.

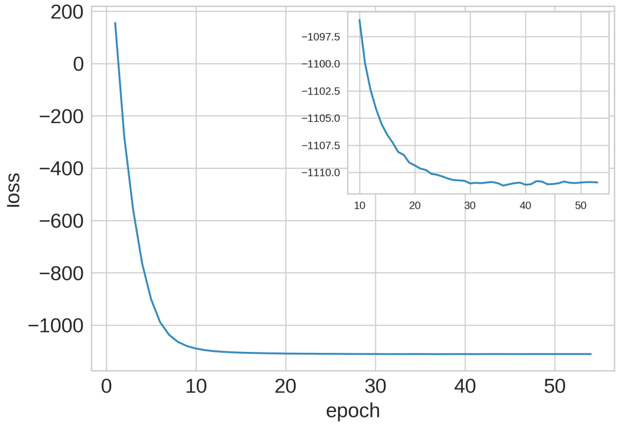

Figure S1 Loss function over training epochs for the synthetic dataset.

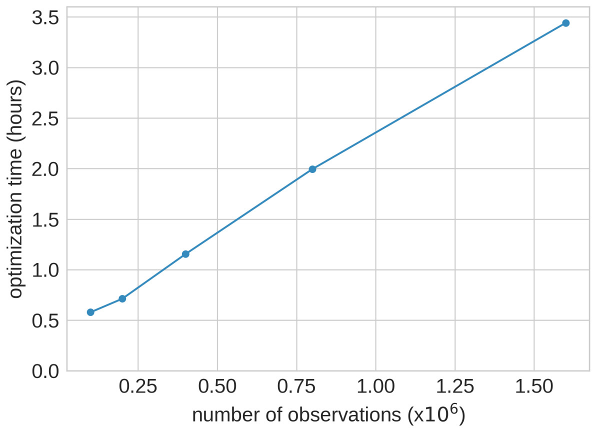

Figure S2 Running time for the optimization of the model for various synthetic data set sizes. For 1.6 million observations the model converges in around 3.5 hours.

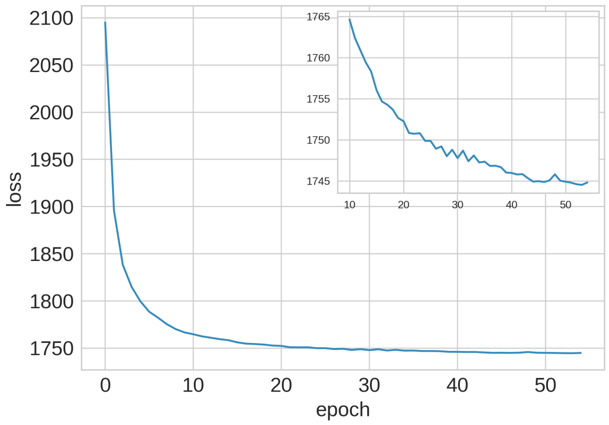

Figure S3 Loss function over training epochs for the wildebeest dataset.

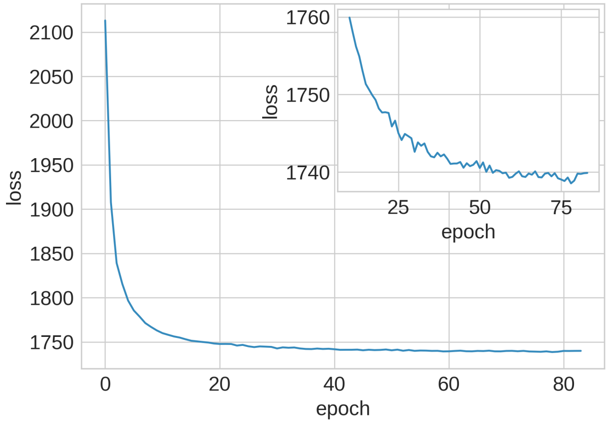

Figure S4 Loss function over training epochs for the wildebeest dataset with environmental covariates.

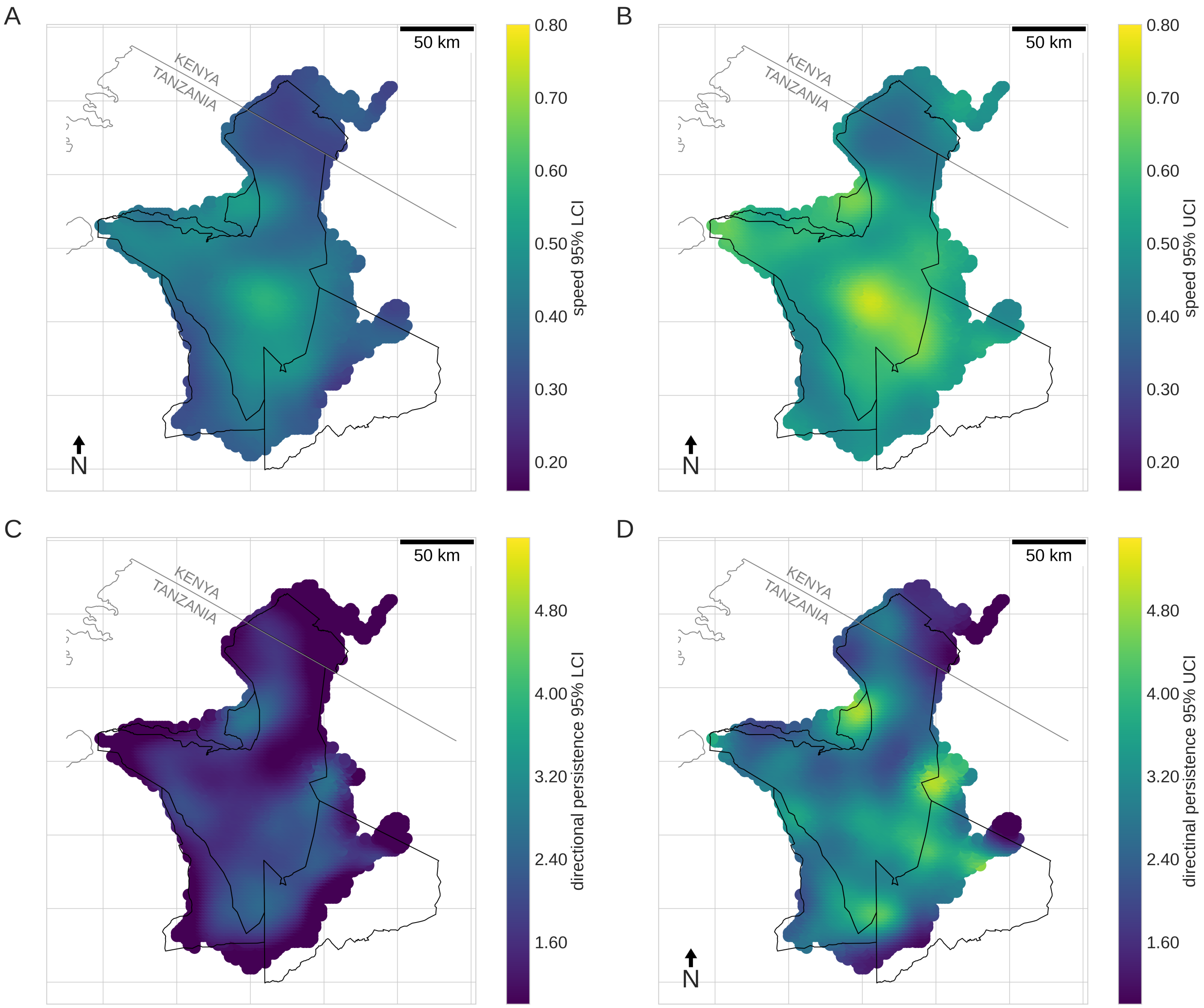

Figure S5 Inference of real environment. (A) and (B) show the lower and upper 95% credible intervals for the average speed. (C) and (D) show the lower and upper 95% credible intervals for the directional persistence.
